# Dietary Phytoestrogens Ameliorate Hydrochloric Acid-Induced Chronic Lung Injury and Pulmonary Fibrosis in Mice

**DOI:** 10.3390/nu13103599

**Published:** 2021-10-14

**Authors:** Pavel Solopov, Ruben Manuel Luciano Colunga Biancatelli, Christiana Dimitropoulou, John D. Catravas

**Affiliations:** 1 Frank Reidy Research Center for Bioelectrics, Old Dominion University, Norfolk, VA 23508, USA; rcolunga@odu.edu (R.M.L.C.B.); cdimitro@odu.edu (C.D.); jcatrava@odu.edu (J.D.C.); 2 School of Medical Diagnostic & Translational Sciences, College of Health Sciences, Old Dominion University, Norfolk, VA 23508, USA

**Keywords:** idiopathic pulmonary fibrosis (IPF), hydrochloric acid, gender differences, phytoestrogens, isoflavones, genistein, mice

## Abstract

We previously reported that female mice exhibit protection against chemically induced pulmonary fibrosis and suggested a potential role of estrogen. Phytoestrogens act, at least in part, via stimulation of estrogen receptors; furthermore, compared to residents of Western countries, residents of East Asian countries consume higher amounts of phytoestrogens and exhibit lower rates of pulmonary fibrosis. Therefore, we tested the hypothesis that dietary phytoestrogens ameliorate the severity of experimentally induced pulmonary fibrosis. Male mice placed on either regular soybean diet or phytoestrogen-free diet were instilled with 0.1 N HCl to provoke pulmonary fibrosis. Thirty days later, lung mechanics were measured as indices of lung function and bronchoalveolar lavage fluid (BALF) and lung tissue were analyzed for biomarkers of fibrosis. Mice on phytoestrogen-free diet demonstrated increased mortality and stronger signs of chronic lung injury and pulmonary fibrosis, as reflected in the expression of collagen, extracellular matrix deposition, histology, and lung mechanics, compared to mice on regular diet. We conclude that dietary phytoestrogens play an important role in the pathogenesis of pulmonary fibrosis and suggest that phytoestrogens (e.g., genistein) may be useful as part of a therapeutic regimen against hydrochloric acid-induced lung fibrosis and chronic lung dysfunction.

## 1. Introduction

Phytoestrogens derived from soy foods have received widespread usage due to their popularized health advantages, including reduced risks of cardiovascular disease and breast and prostate cancers and alleviation of menopause-related symptoms [1]. Most of these benefits are attributed to the presence of isoflavones [2], such as genistein, daidzein, and glycetein, the most studied group of diphenolic compounds that are classified more accurately as selective estrogen receptor (ER) modulators (SERM); they bind with low affinity to both types of ER, but tend to have a higher affinity for ERβ [3,4,5]. The presence of ERα is also obligatory for transcriptional activity, which is triggered by the SEERM-ERβ link, probably via the formation of ERα/ERβ heterodimers [6]. Soy is one of the most common sources of protein in the majority of commercial formulas for laboratory rodent diets, thus, soy-based animal diets may influence estrogen-regulated systems [2]. The content of phytoestrogens in rodent diets depends on several factors, but primarily on the soy content of the diet [7]. Isoflavone content of soybeans may vary, in some cases several fold, depending on soy variety and growth conditions, such as type and quality of the soil, temperature, duration of the humidity season, and daylight hours [8]. These variables help explain both the differences between commercial diet brands, and the large batch-to-batch variations in nutrition [7]. Thus, the content of soy in laboratory animal diets may significantly affect the outcome of sex-related diseases, such as idiopathic pulmonary fibrosis (IPF), a devastating disease, characterized by the progressive substitution of the lung parenchyma with fibrotic tissue, and associated to poor prognosis with an expected mean survival of up to five years from the time of diagnosis [9].

Mortality from pulmonary fibrosis has been increasing. Only in the USA, more than 100 deaths per 100,000 population occur every year [10]. IPF is more prevalent among males than females [11,12], but very little is known about sex-related differences in the presentation of the disease and associated comorbidities. In a multidimensional indexing and scoring system for Idiopathic PF patients, the Gender-Age-Physiology score, men show a higher risk of dying compared to women [13], but the mechanisms leading to this are still poorly characterized. A possible cause of gender inequality in mortality data may be the protective role of estrogens. In countries of East Asia and South America, where traditionally the consumption of soy products is higher (~50 mg isoflavones per day), the incidence of Idiopathic Pulmonary Fibrosis (IPF) is lower than in Western countries (0.15–3 mg isoflavones per day) [14,15,16,17].

We recently reported on sex-related pathways involved in the fibrotic process of the lung [18]. In this study we investigate whether a diet poor in ER-stimulating phytoestrogens affects the development of PF in a well-characterized mouse model of HCl-induced lung injury and pulmonary fibrosis [19].

## 2. Materials and Methods

### 2.1. Materials

Teklad Global Rodent Diets 2018 and 2020X were purchased from Envigo (Indianapolis, IN, USA). Hydrochloric acid (ACS grade), methacholine (USP grade), red protein G affinity gel beads, RIPA lysing buffer, and protease inhibitor cocktail were obtained from Sigma-Aldrich Corporation (St. Louis, MO, USA). Socumb (pentobarbital) USP grade, AnaSed (xylazine) USP grade, and Ketaset (ketamine) USP grade were supplied by Henry Schein Animal Health (Pittsburg, PA, USA). Ten percent formaldehyde, PureLink^TM^ DNase Set, RNase Inhibitor, and RNAlater were purchased from Thermo Fisher Scientific (Waltham, MA, USA); BCA Protein assay kit from Pierce Co. (Rockford, IL, USA); EDTA and nitrocellulose membranes from GE Healthcare (Chicago, IL, USA); TRIzol^®^ and SuperScript^TM^ IV VILO Reverse transcription Kit were from Invitrogen (Carlsbad, CA, USA); Neasy mini kit from Qiagen (Hilden, Germany); and SYBR Green Master Mix from Applied Biosystems (Carlsbad, CA, USA). Fibronectin, elastin, and beta-actin primers used for real time quantitative PCR were purchased from Integrated DNA technologies, Inc. (Coralville, IA, USA). All antibodies used in immunoblotting have published immunospecificity data available online. The following antibodies used in Western blots: rabbit total (#5339S) and phosphorylated SMAD2 (#18338S) and HSP90 (#3488S) were obtained from Cell Signaling Technology, Inc. (Danvers, MA, USA); mouse monoclonal anti-β-Actin (#A5441) from Sigma-Aldrich Corporation, Collagen IA2 rabbit antibody (#PA5-50938) from Thermo Fisher Scientific (Waltham, MA, USA); IRDye 800CW goat anti-rabbit (#D10121-05) and IRDye 680RD goat anti-mouse (#C90910-21), NewBlot PVDF Stripping Buffer from LI-COR Biosciences (Lincoln, NE, USA). For preparation of SDS-PAGE: ProtoGel (30% acrylamide mix) and TEMED were from National Diagnostics (Atlanta, GA, USA), Tris-HCl buffer from Teknova (Hollister, CA, USA); 10% SDS and ammonium persulfate from Thermo Fisher Scientific; protein dual color standards and Tricine Sample Buffer were purchased from Bio-Rad Laboratories.

### 2.2. Animals and Treatment Groups

All animal studies were approved by the Old Dominion University IACUC and adhere to the principles of animal experimentation as published by the American Physiological Society. Healthy male C57Bl/6J mice (Jackson Laboratories, Bar Harbor, ME, USA), 8–10 weeks old, 25–28 g body weight, were randomly separated into three experimental groups: (1) Vehicle group: mice on “regular” Teklad Global Rodent Diets 2018 diet (content of isoflavones 150–340 mg/kg), received 2 μL/g body weight saline intratracheally (i.t.); (2) HCl regular diet group: mice on the same soybean based diet, received 2 μL/g body weight of 0.1 N HCl, i.t.; (3) Phytoestrogen-free diet HCl group: mice on Teklad Global Rodent Diet 2020X (content of isoflavones <20 mg/kg) starting 2 weeks before exposure to 2 μL/g body weight of 0.1 N HCl, i.t.; (*n*  =  12 mice per group). The composition of the other nutrients of both diets is similar. To intratracheally instill HCl or saline, mice were anesthetized with intraperitoneal (i.p.) injections of AnaSed (xylazine, 6 mg/kg) and Ketaset (ketamine, 60 mg/kg). An intraperitoneal injection of sterile saline (10 μL/g) was given as pre-emptive fluid resuscitation. A 1 cm neck skin incision and blunt dissection of the salivary glands was made to visualize the trachea, and while mice were suspended vertically, a fine 20 G plastic i.v. catheter was introduced into the trachea through the mouth; cannulation of the trachea was confirmed by visualization of the catheter from the open neck incision. Then, freshly prepared HCl solution (groups 2 and 3) or sterile saline (group 1) was instilled (2 µL/g) and flushed with ~100 μL air. The catheter was then withdrawn, the neck incision was closed by VetBond surgical adhesive, and the animals were placed in ventral position on top of a heating pad, under supplemental oxygen (slowly weaned from 100 to 21% O_2_), and observed for the next few hours for signs of respiratory distress. Mice were later returned to their home cages and monitored daily for abnormal physical appearance. ll analyses were performed at 30 days post i.t. instillation.

### 2.3. Lung Mechanics Measurements

Thirty days after intratracheal instillation, all mice were anesthetized with Socumb (pentobarbital 90 mg/kg, i.p.), tracheostomized with a metal 1.2 mm (internal diameter) cannula, and connected to a FlexiVent small animal ventilator (SCIREQ Inc., Montreal, QC, Canada). Ventilation was performed at a tidal volume of 10 mL/kg and respiratory rate of 150/min. A 15-min stabilization period was allowed before any measurements began. Following a deep inflation, resting static compliance (Cst, mean of 3 values) and pressure-volume relationships (PV curves) were estimated by stepwise increasing airway pressure to 30 cm H_2_O and then reversing the process. Both parameters reflect the intrinsic elasticity of the lungs and are either reduced (Cst) or shifted to the right (PV curves) in fibrosis. Secondly, Snapshot-150 and Quick Prime-3 maneuvers were performed. Respiratory system resistance (Rrs) and elastance (Ers), reflecting the behavior of the entire respiratory system (peripheral and conducting airways, chest wall, and parenchyma), and Newtonian resistance (Rn) and tissue damping (G) values, the former reflecting resistance of the large, conducting airways, and the latter reflecting mostly parenchymal and peripheral airway contributions, were calculated, and are presented as the mean of at least 12 recordings for each animal.

### 2.4. Histopathology and Lung Injury Scoring

Immediately after euthanasia, chest was open, the lungs were fixed with 10% formaldehyde, embedded in paraffin and were stained with hematoxylin and eosin (H&E) and, for collagen staining, with Masson’s trichrome, as we previously described [18]. Twenty randomly selected fields from each slide were examined under 20, 40, and 100× magnifications. Fields were scored according to the Lung Injury Score [20] and Ashcroft score [21] methods by an investigator blinded to the identity of the study groups.

### 2.5. Bronchoalveolar Lavage Fluid (BALF) White Blood Cells Count

Bronchoalveolar lavage fluid (BALF) was collected as described before [18]. The total number of white blood cells was determined using a hemocytometer.

### 2.6. Total Protein and Cytokines Analysis in BALF

BALF supernatant was collected and prepared as described above. The protein concentration was determined using the micro bicinchoninic acid (BCA) assay according to the manufacturer’s protocol. BALF supernatant TGF-β1 was analyzed in triplicate via a mouse/human TGF-β1 ELISA kit.

### 2.7. Lung Tissue Collection

The lungs were dissected from the thorax, snap-frozen, and prepared for subsequent analysis as we previously described [18].

### 2.8. Western Blot Analysis

Proteins in lung tissue homogenates were extracted from frozen lungs by ultrasonic homogenization (50% amplitude, 3 times for 10 s) in ice-cold lysing RIPA buffer with added protease inhibitor cocktail (100:1). The protein lysates were gently mixed under rotation for 3 h at 4 °C, and then centrifuged twice at 14,000× *g* for 10 min at 4 °C. The supernatants were collected, and total protein concentration was analyzed using the micro-BCA assay. Equal amounts of proteins from all samples were used for Western blot analysis. The lysates were first mixed with Tricine Sample Buffer 1:1, boiled for 5 min, and then separated on a 10% polyacrylamide SDS gel by electrophoresis. Separated proteins were then transferred to a nitrocellulose membrane, incubated overnight at 4 °C with the appropriate primary antibody, diluted in the blocking buffer, followed by one hour incubation with the secondary antibody at room temperature and scanned by digital fluorescence imaging (LI-COR Odyssey CLx, Dallas, TX, USA). βactin was used as housekeeping protein. ImageJ software v.1.8.0 was used to quantify the bands from the Western blot membranes (http://imagej.nih.gov/ij/, accessed on 15 May 2021);National Institutes of Health, Bethesda, MD, USA). Some membranes were stripped for 5 min and incubated with other primary and secondary antibodies.

### 2.9. RNA Isolation and Quantitative Real-Time PCR (qPCR)

Lung tissue, stored in RNAlater solution for at least 24 h, was dried and homogenized in TRIzol^®^ followed by a cleaning-up step using the RNeasy Mini Kit. The purified RNA was transcribed into cDNA using the SuperScriptTM IV VILO Reverse transcription Kit and analyzed by real-time qPCR with SYBR Green Master Mix on a StepOne Real-Time PCR System (Applied Biosystems v.2.3). Results were evaluated using the standard curve method and expressed as fold of control values. βactin mRNA expression was used for the normalization of each mRNA expression levels for all samples.

### 2.10. Statistical Analysis

Statistical significance of differences among groups was determined by one-way or two-way analysis of variance (ANOVA) followed by the Tukey post-hoc test using GraphPad Prism Software (GraphPad Software, San Diego, CA, USA). Differences among groups were considered significant at *p* < 0.05.

## 3. Results

### 3.1. Phytoestrogen-Deficient Diet Aggravates HCl-Induced Mortality

**Figure 1:**
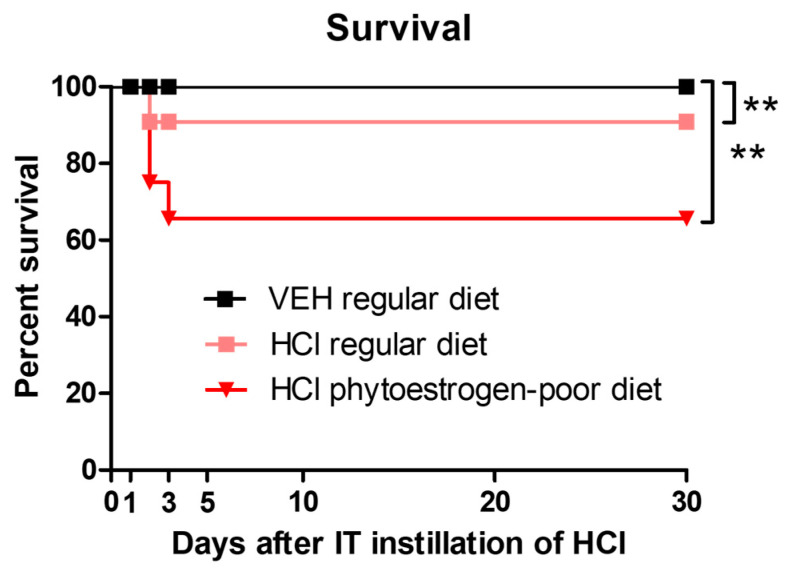
Mice on normal diet are more resistant to HCl-induced mortality. Kaplan–Meyer survival curves. Means ± SEM; **: *p* < 0.01, with ANOVA and Tukey’s, *n* = 6–8; VEH: Vehicle.

We observed a mortality rate of 0 and 9% for mice on regular diet exposed to saline or 0.1 N hydrochloric acid, respectively. Mice on phytoestrogen-free diet that received HCl exhibited significantly higher mortality, 38% (Figure 1).

### 3.2. Dietary Phytoestrogens Reduce HCl-Induced Chronic Alveolar Inflammation

**Figure 2:**
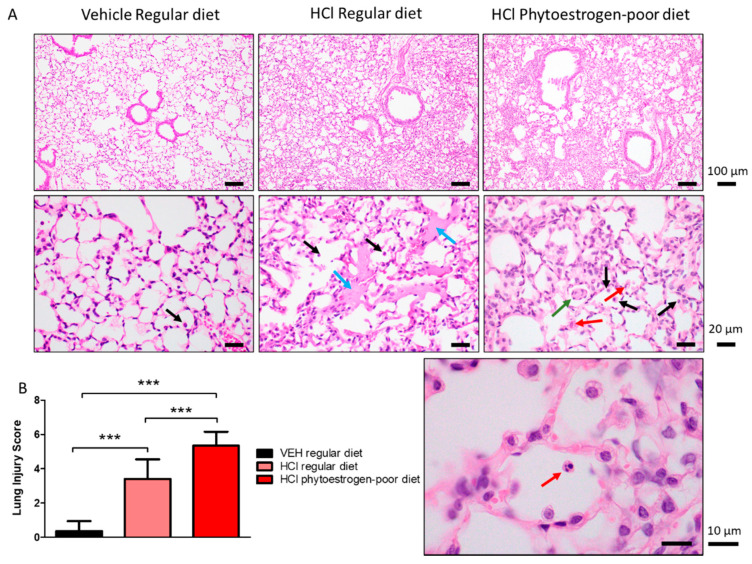
HCl causes chronic lung injury. (**A**) H&E staining of lung sections demonstrates monocyte infiltration and hyaline membranes in regular diet-fed, HCl-instilled mice, edema, septal thickening, monocyte, and neutrophil infiltration in isoflavone-poor diet-fed mice. (**B**) Lung Injury Score is maximal in HCl-instilled mice, on phytoestrogen-poor diet, still high but lower in HCl-instilled mice on regular diet and without significant changes in saline-instilled mice. (Means ± SEM; *n* = 3; ***: *p* < 0.001; with one-way ANOVA and Tukey’s); VEH: Vehicle.

Animals instilled with vehicle showed no signs of alveolar inflammation (Figure 2A). Mice on regular diet and instilled with HCl demonstrated thickening of alveolar walls, infiltration of alveolar and interstitial mononuclear cells (black arrows), and massive formation of hyaline membranes (blue arrows) in the alveolar space.

HCl-instilled mice on isoflavone-poor diet showed more severe signs of lung injury (Figure 2A,B). In addition to monocytes (black arrows), neutrophiles were observed to infiltrate the alveolar spaces (red arrows), as was granuloma formation inside the alveolus (green arrows).

**Figure 3:**
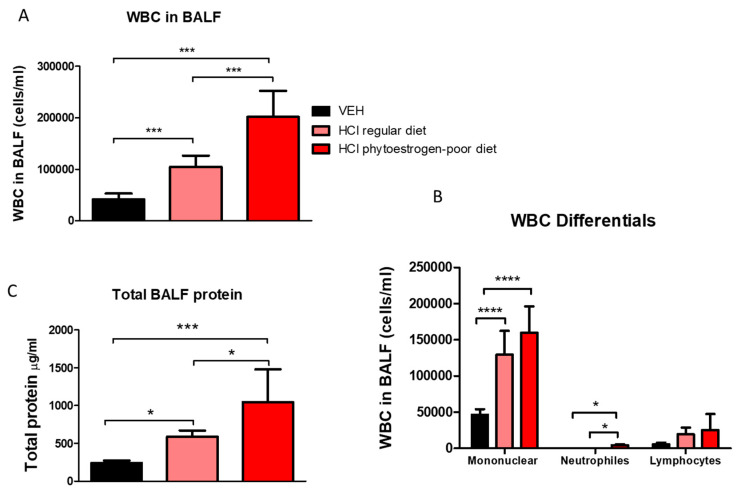
White blood cell (WBC) content (**A**), differential WBC content (**B**), and total protein concentration (**C**) in bronchoalveolar lavage fluid (BALF) 30 days after instillation of HCl or saline (Means ± SEM; *n* = 8–9; ****: *p* < 0.0001; ***: *p* < 0.001, *: *p* < 0.05 with one-way ANOVA and Tukey’s; VEH: Vehicle.

Moderate alveolar inflammation was also reflected in increased concentrations of leucocytes and proteins in BALF of HCl-instilled mice on phytoestrogen-poor diet, and significantly less in HCl-instilled mice on normal diet, whereas baseline values were observed in mice on regular diet receiving saline (Figure 3A). A similar trend was observed in total protein concentration with a significant increase in both groups instilled with 0.1 N HCl, but substantially more in mice on phytoestrogen-poor diet (Figure 3C). Following acid instillation, the proportion of monocytes and alveolar macrophages increased in mice of both groups, while the level of neutrophils was significantly higher in the phytoestrogen-poor group (Figure 3B).

### 3.3. Phytoestrogen-Deficient Diet Aggravates HCl-Induced Pulmonary Fibrosis

**Figure 4:**
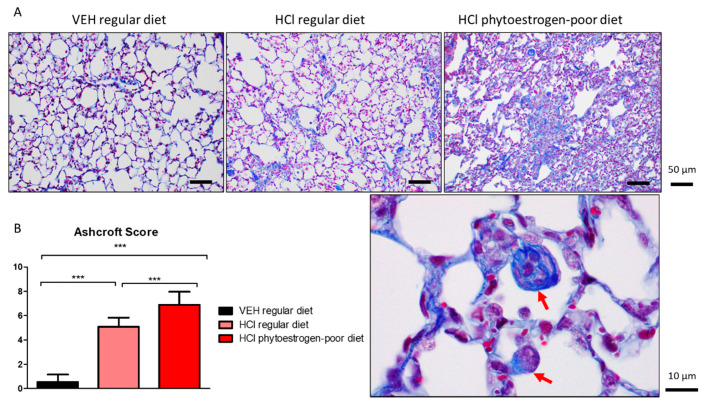
HCl induces pulmonary fibrosis. (**A**) Masson’s Trichrome staining of lung sections demonstrates moderate collagen deposition in regular diet-fed HCl-instilled mice and total fibrous obliteration, and loss of parenchymal architecture in isoflavones-poor diet-fed mice. (**B**) The Ashcroft score depicts severe fibrosis in HCl-instilled mice on phytoestrogen-free diet, a milder pathology in HCl-instilled mice on regular diet and no significant changes in saline-instilled mice. (Means ± SEM; *n* = 3; ***: *p* < 0.001; with one-way ANOVA and Tukey’s); VEH: Vehicle.

At 30 days after hydrochloric acid instillation, increased parenchymal collagen deposition was observed in mice on a regular diet. Progressive collagen deposition with fibrotic areas was localized along the alveolar walls; however, the parenchymal architecture was relatively conserved. Tissues from mice on isoflavones-poor diet, showed major histological alterations, including large areas with total fibrous obliteration. In the infrequently open alveolus, collagen appears to envelop alveolar macrophages (Figure 4, red arrows).

### 3.4. Dietary Phytoestrogens Modulate HCl-Induced Activation of TGF-β Signaling and Expression of Extracellular Matrix Proteins

**Figure 5:**
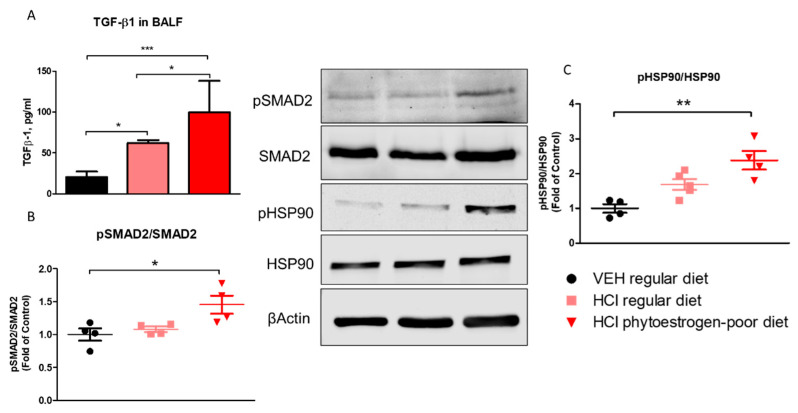
TGF-β levels and activation of intracellular pathways of TGF-β 30 days after HCl instillation. (**A**) Mice treated with HCl displayed increased expression levels of TGF-β1 in bronchoalveolar lavage fluid (BALF) compared to saline. The levels of TGF-β1 in mice on phytoestrogen-poor diet is significantly higher compared to mice on regular diet. (**B**) Active (phosphorylated) SMAD2 was significantly increased in mice on isoflavone-poor diet but not in animals on regular diet. (**C**) Heat Shock Protein 90 activation (pHSP90) increased only in phytoestrogen-poor fed mice. Means ± SEM; ***: *p* < 0.001, **: *p* < 0.01, *: *p* < 0.05 with one-way ANOVA and Tukey’s, *n* = 8–9 (**A**), *n* = 4–5 (**B**,**C**); VEH: Vehicle.

Mice on phytoestrogen-poor diet and instilled with HCl demonstrated higher expression levels of TGF-β1 than animals receiving the regular diet or saline controls (Figure 5A). The canonical SMAD signaling pathway of TGF-β, analyzed in lung homogenates, showed increased activated levels of SMAD2 in mice receiving isoflavone-poor diet, while HCl-instilled animals receiving a regular diet did not show significant changes (Figure 5B). A similar pattern was observed with the activation (phosphorylation) of Heat Shock Protein 90 (HSP90), a crucial pro-fibrotic chaperone (Figure 5C).

**Figure 6:**
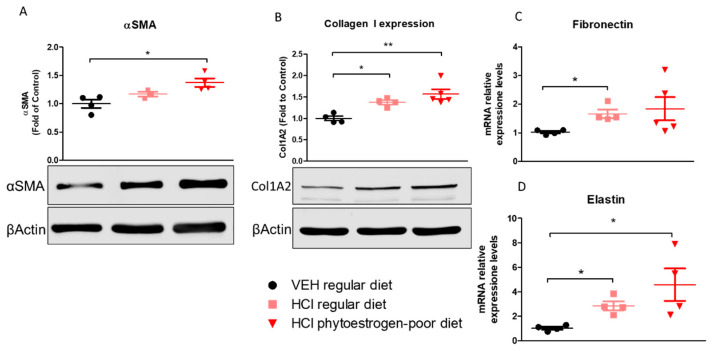
Extracellular matrix protein or mRNA expression in lung tissue. α-Smooth Muscle Actine (**A**), Collagen Type I (**B**), Fibronectin (**C**) and Elastin (**D**) were overexpressed in mice on phytoestrogen-poor diet. Means ± SEM; **: *p* < 0.01, *: *p* < 0.05 with one-way ANOVA and Tukey’s, *n* = 45; VEH: Vehicle.

Lung alpha-smooth muscle actin (αSMA) was significantly increased in phytoestrogen-poor-fed, HCl-instilled mice, but not in mice on regular diet (Figure 6A). Collagen Type I levels increased in both HCl-instilled groups compare to control. A similar pattern was observed with elastin mRNA levels. Fibronectin, one more key extracellular matrix protein, showed significant increase (mRNA) only in lungs from mice on regular diet.

### 3.5. Dietary Phytoestrogens Protect against HCl-Induced Lung Dysfunction

**Figure 7:**
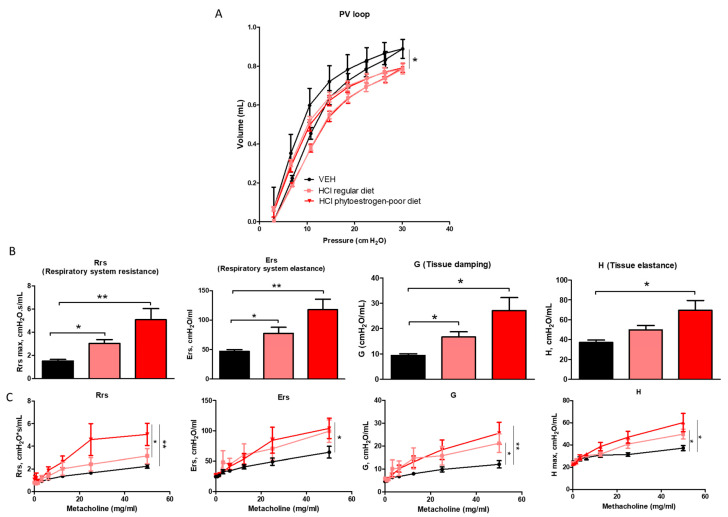
Effect of dietary phytoestrogens on HCl-induced alterations in lung mechanics. Phytoestrogen diet had a significant impact on preventing a downward shift of pressure volume (PV) loops after HCl instillation (**A**). Respiratory system resistance (Rrs), elastance (Ers), tissue elastance (H), and damping (G) increased, compared to control mice in all groups instilled with HCl (**B**,**C**). Means ± SEM; *n* = 6 mice per group; *: *p* < 0.05, **: *p* < 0.01, with one- or two-way ANOVA and Tukey’s; VEH: Vehicle.

Changes in lung mechanics were also evident among different diet groups after HCl instillation (Figure 7). Respiratory system resistance (Rrs), respiratory system elastance (Ers), tissue damping (G), and tissue elastance (H) increased significantly in HCl-instilled mice on either regular or isoflavone-poor diet when compared to saline controls. However, animals on phytoestrogen-free diet demonstrated a more dramatic increase compared to controls and a significant increase compared to the regular diet group. A similar pattern was observed in response to increasing concentrations of aerosolized methacholine. Moreover, pressure–volume (PV) loops of HCl-instilled isoflavones-poor-fed mice showed a characteristic downward shift reflecting stiffer lungs, whereas PV loops of mice on regular diet displayed a lesser shift.

## 4. Discussion

Most laboratory rodent diets include soy proteins that provide large dosages of isoflavones to animals throughout their life, starting from the perinatal period. Although it is well known that isoflavones are consistently high in the serum and urine of people for whom soy foods are a main part of their diet, it is often neglected that commercial rodent diets drafted with soy meal cause rodents to also exhibit constant high steady-state serum isoflavone concentrations [22]. In this study, we investigated how the lack of isoflavones in rodent diet can affect the development of pulmonary fibrosis associated with a single exposure to HCl. Sex-dependent variances in PF have been suggested by several investigators [23,24]. These differences stem from a higher prevalence of disease and higher mortality in males [25]. There are many studies suggesting that phytoestrogens increase survival from cardiovascular deceases [26] and cancers [27,28,29]. A limited number of studies suggest the impact of nutrition to the development of lung diseases. In an epidemiological study, a high intake of saturated fat was associated with an increased risk of IPF. [30]. Both regular and phytoestrogen-free diets used in our study have similar content of fat (6.2–6.5%). A number of animal studies have reported that many nutrients (including polyphenols) can exert a beneficial or detrimental actions on the progression of lung fibrosis [31]. In a previous study, we demonstrated the sex-dependence of fibrogenesis in two mice models of PF [18]. Here, for the first time, we show that phytoestrogen-poor diet could significantly enhance the mortality of animals exposed to HCl.

Initially, isoflavones were thought to act as anti-inflammatory agents because of their down-regulation of cytokine-induced signal transduction facts in immune cells [32]. Subsequently, an increasing number of studies have substantiated that isoflavones exhibit anti-inflammatory effects. Isoflavones scavange a wide range of reactive oxygen, nitrogen, and chlorine species, and are relatively resistant to oxidation mediated by strong oxidants such as hypochlorous acid and peroxynitrite [33]. Oral administration of isoflavones or of extracts of soy products decrease serum nitrite, nitrate, and nitrotyrosine levels in lipopolysaccharide-(LPS)-treated rats [34]. In mouse models, the isoflavone genistein demonstrates anti-inflammatory effects, which are reflected in reduced granulocyte and mononuclear leukocyte content [35]. In a guinea pig model of asthma, genistein significantly inhibited ovalbumin-induced acute bronchoconstriction, reduced pulmonary eosinophilia and eosinophil peroxidase activity [36]. We recently demonstrated that HCl causes endothelial barrier dysfunction in human lung microvascular endothelial cells [37], which supports the increase in total BALF protein observed here. In this study, we confirmed the anti-inflammatory effect of dietary isoflavones. Mice on a regular diet with high isoflavone content demonstrated lower HCl-induced alveolar inflammation, lower total cell count in BALF, particularly monocytes and neutrophils, improved endothelial barrier function, and had less vascular permeability, compared to mice on isoflavone-poor diet.

**Figure 8:**
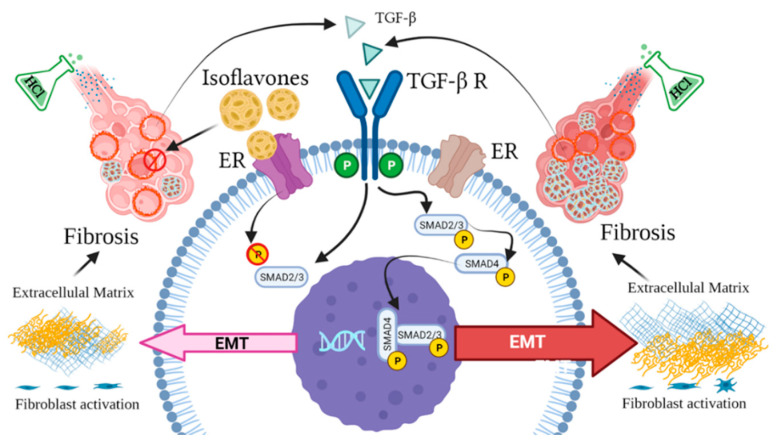
Estrogen-dependent signaling pathways mediating HCl-induced pulmonary fibrosis. Dietary phytoestrogen isoflavones modulate alveolar inflammation and block TGF-β SMAD signaling through activation of estrogen receptor (ER), preventing fibroblast activation and overexpression of extracellular matrix.

There is increasing data suggesting the potential benefits of isoflavones as antifibrotic agents. For example, stellate cells express the beta but not the alpha isoform of the estrogen receptor, and nutritional intake of the soy isoflavone genistein—a selective agonist of ERβ at low nanomolar plasma concentrations that are achievable with such intake—can suppress liver fibrosis, in both genders [38]. Further, genistein significantly ameliorated myocardial fibrosis in rats [39] and soy isoflavones reduced the vascular damage, inflammation, and fibrosis caused by radiation damage to lung tissue in mice [40]. Radiation-challenged rats, treated with genistein, showed significant decreases in hydroxyproline and in levels of activated macrophages in lung tissue [41]. We and others have previously provided evidence implicating HSP90 in lung fibrogenesis [37,42,43,44,45,46]. In the present study, we also report activation of HSP90 in mice receiving phytoestrogen-poor diet. Also, for the first time, we demonstrate the possible ability of dietary phytoestrogens to moderate pulmonary fibrosis, as reflected in the fibrotic score and in the activation of the canonic SMAD-signaling TGF-β pathway (Figure 8). Genistein has the ability to block α-SMA and inhibit connective tissue growth factor (CTGF) expression in human renal tubular epithelial cells [47]. The isoflavone puerarin regulates the expression of TGF-β1 and α-SMA in alcohol-induced liver fibrosis in rats [48]. In the present study, mice on isoflavone-poor diet demonstrated significant increases in α-SMA, collagen I and elastin deposition. The decrease in extracellular matrix protein release could be explained by a likely decrease in estrogen receptor signaling due to lack of phytoestrogen-mediated ER activation. During menopause, the decrease in circulating estrogen levels causes dysfunctions of the connective tissues via ECM degradation [49]. Laboratory animals probably become adapted to high-phytoestrogen intake over many generations, eating soy-based diets, and removing all phytoestrogens from the diet most likely leads to readjustments that could disrupt multiple biological functions [50].

Phytoestrogens could improve lung function damage following HCl exposure. Genistein has been shown to attenuate ovalbumin-induced airway hyperresponsiveness to inhaled methacholine in asthmatic guinea pigs [36]. Here we also observed the significant deterioration of respiratory resistance, elastance, and tissue damping in mice receiving phytoestrogen-poor diet compared to mice receiving diet with isoflavone content.

## 5. Conclusions

This study demonstrated that dietary isoflavones reduce pulmonary fibrosis and ameliorates lung function in mice exposed to HCl. Our data suggests that isoflavone content is of great importance to the diet. Soy isoflavone genistein could be a novel and effective strategy for antifibrotic supplementation in people at risk of exposure to HCl.

## Data Availability

Not applicable.
